# The Impacts of Social Media Use and Online Racial Discrimination on Asian American Mental Health: Cross-sectional Survey in the United States During COVID-19

**DOI:** 10.2196/38589

**Published:** 2022-09-19

**Authors:** Alyan Layug, Samiksha Krishnamurthy, Rachel McKenzie, Bo Feng

**Affiliations:** 1 Department of Asian American Studies University of California, Davis Davis, CA United States; 2 Department of Psychology University of California, Davis Davis, CA United States; 3 Department of Communication University of California, Davis Davis, CA United States

**Keywords:** Asian Americans, mental health, COVID-19 pandemic, racial discrimination, social media, anxiety, depression, secondary traumatic stress, negative affect, racial/ethnic identification

## Abstract

**Background:**

During the COVID-19 pandemic, increased social media usage has led to worsened mental health outcomes for many people. Moreover, due to the sociopolitical climate during the pandemic, the prevalence of online racial discrimination has contributed to worsening psychological well-being. With increases in anti-Asian hate, Asian and Asian American social media users may experience the negative effects of online racial discrimination in addition to the reduced psychological well-being resulting from exposure to online COVID-19 content.

**Objective:**

This study aims to investigate the impact of COVID-19–related social media use and exposure to online racial discrimination during the pandemic on the mental health outcomes (ie, anxiety, depression, and secondary traumatic stress [STS]) of Asian Americans compared with those of non-Asian Americans. In addition, this study explores the mediating role of negative affect and the moderating role of racial/ethnic identification.

**Methods:**

An online survey was conducted through Amazon Mechanical Turk and a university-wide research portal from March 3 to March 15, 2021. A total of 1147 participants took the survey. Participants’ social media usage related to COVID-19 and exposure to 2 online forms of racial discrimination (individual and vicarious), mental health outcomes (anxiety, depression, and STS), racial/ethnic identification, negative affect, and demographics were assessed.

**Results:**

Our results showed that COVID-19–related social media use, individual discrimination, and vicarious discrimination were predictors of negative mental health outcomes (anxiety, depression, and STS). Asian Americans reported higher vicarious discrimination than Latinx and White Americans, but Asian Americans’ mental health outcomes did not differ substantially from those of the other racial/ethnic groups. Racial/ethnic identification moderated the relationship between both types of discrimination and STS, and negative affect served as a mediator between both types of discrimination and all 3 mental health outcomes.

**Conclusions:**

These results suggest that social media exposure continues to have a dire effect on mental health during the COVID-19 pandemic. This study helps to contextualize the rise of anti-Asian American hate and its impact on mental health outcomes in the United States.

## Introduction

### Background

Because of a surge in social media usage by millions of individuals during the COVID-19 pandemic, social media has played an important role in the communication of disaster and health crisis–related news [1-4]. Social media may provide forms of comfort and mutual connectivity during phases of social distancing [5] and assist in sharing social, psychological, and instrumental aid [6], as well as up-to-date information regarding the pandemic [7,8]. Meanwhile, it is on social media platforms such as Twitter, Instagram, and Facebook where individuals constantly engage with disaster and health crisis–related information, such as news of deaths and mass paranoia regarding COVID-19 [9,10]. Research has shown that disaster-related content on social media during events of collective trauma (such as the COVID-19 pandemic) may negatively impact users’ mental health [11-13]. Additionally, racialized hate incidents that have occurred on the internet may exacerbate the negative influence of the pandemic on racial/ethnic minority groups’ mental health [14,15]. To date however, little research has conducted an integrated and contextualized empirical investigation of how the use of certain media with regard to specific types of content during significant historical and public health contexts can exert differential impacts on different groups of people, as well as the mechanisms underlying the impacts. This type of investigation is especially important, given the mixed findings of media effects documented in prior research, and can provide a nuanced understanding of the complexity of the phenomenon.

This study, therefore, aims to fill this gap. More specifically, we investigate the impact of social media usage related to COVID-19 content and social media exposure to racial discrimination on the mental health outcomes of Asian Americans (defined in this paper as anyone of Asian ethnicity living in the United States regardless of nationality) and various other racial/ethnic groups in the United States. We also examine the mediating role of negative affect and the moderating role of racial/ethnic identification in these processes.

### Social Media Usage Related to COVID-19

Social media refers to a group of internet-based applications that allow for the creation and exchange of user-generated content and enable users to participate in online social networking [16-18]. For the purposes of this study, we specifically delineated social media to include SMS text messaging, online chats, comment sections, discussion forums, online games, and social networking sites (eg, Yubo, Instagram, Twitter, and TikTok). Social media usage covers a broad range of online activities, and scholars have categorized these activities into the following two primary types: active and passive use [19-21]. Active use relates to a conscious behavior to share information [22]. This includes activities such as posting, commenting, and messaging. By contrast, passive use involves the consumption of content rather than the creation of content (eg, browsing). While scrolling or browsing content passively, social media users may have little control over what is presented to them. This is seen as “lurking” on the internet and has been shown to be the most prominent form of usage on social media sites [23].

In this study, social media use related to COVID-19 refers to any form of social media use related to COVID-19 or the COVID-19 pandemic (eg, changes to public safety measures or a Facebook friend sharing their experience with COVID-19) that appears online, including on social media platforms. In the context of the COVID-19 pandemic, social media usage related to COVID-19 has been shown to be associated with poorer psychological outcomes [14]. Chao et al [24], for example, found that Chinese individuals’ use of social media during the pandemic was positively associated with negative affect, anxiety, and stress. Social media usage predicted depression and secondary traumatic stress (STS; defined as emotional duress that results when an individual hears about the firsthand trauma experiences of another) among Wuhan residents in early February 2020, 2 weeks after Wuhan was put on lockdown [25]. A national study conducted across 30 provinces in China found that social media usage related to COVID-19 content mediated the levels of traumatic emotions among nonpatients, but did not cause negative mental health symptoms nor lead to a negative impact on mental health [26]. Furthermore, in studies on China’s netizens, social media exposure to COVID-19–related content was found to be associated with poor mental health outcomes such as anxiety and depression [9]. However, to our knowledge, no current research has investigated the impact of COVID-19–related social media usage on mental health outcomes in the United States. Although Riehm et al [27] found a positive association between media exposure and mental distress in a sample of adults living in the United States during the beginning of the pandemic, they did not focus specifically on COVID-19 social media content. In accordance with the current research on social media usage and the pandemic, we predict that individuals’ social media usage related to COVID-19 content would be positively associated with their anxiety, depression, and STS.

### Exposure to Online Discrimination

Exposure to online racial discrimination is an added stressor for racial/ethnic minority groups in the United States, especially during the COVID-19 pandemic [28-30]. Online racial discrimination is defined in this study as any form of discrimination denigrating or excluding individuals or groups on the basis of race through the use of symbols, voice, video, images, text, and graphic representations [31]. Online racial discrimination can be broken down into 2 forms: individual online racial discrimination and vicarious online racial discrimination, referred to as individual and vicarious discrimination, respectively, hereafter. Individual online discrimination is any form of racialized discrimination that is directly targeted at and perceived by an individual online. By contrast, vicarious online discrimination is the secondhand exposure to online racial discrimination or prejudice directed at an individual’s community [32,33]. Research has shown that both individual and vicarious discrimination are significantly and positively associated with psychological distress and negative behavioral patterns such as alcohol use [34,35].

Studies have shown that various racial groups have experienced discrimination online due to their race or ethnicity [36-40], and this discrimination has continued throughout the COVID-19 pandemic [41-43]. During the COVID-19 pandemic, Asian Americans and people of Asian ethnic origin have become particularly vulnerable to racial discrimination. Asian Americans throughout US history have been and continue to be subjected to forms of xenophobia, hate, and bias [41-43], dating back to the concept of Orientalism, which refers to viewing Asians through a Western lens consisting of exoticism and a sense of superiority over the “inferior” Eastern countries. Discrimination against Asian Americans has dramatically risen in the past couple of years due to the link that has been drawn between COVID-19 and Wuhan, China, through multiple sources, especially the mass media [44]. Coinciding with this rhetoric, a large body of recent research has revealed how Asians specifically have been the subject of hate, cruelty, and anger after the outbreak of the COVID-19 pandemic [43-50]. During the start of the pandemic from March 19, 2020, to May 13, 2020, more than 1700 Anti-Asian hate incidents were reported to Stop Asian, Pacific Islander, and Mixed Asian (AAPI) Hate nationwide [51].

Regardless of their pan-ethnic Asian identities (eg, South Asian, Filipino, Japanese, and Hmong), Asians have been targets of derogatory language and attacks on public social media platforms since the beginning of the pandemic [45,52]. Stop AAPI Hate’s national report through June reveals that Asian Americans experienced a sharp increase in online hate from 2020 (6.1%) to 2021 (10.6%) [53]. In addition, as outlined by Cheah et al [14], Chinese Americans have experienced both individual and vicarious forms of online COVID-19–related racial discrimination. Because of the sharp increase in anti-Asian online sentiments, it can be reasonably inferred that Asian Americans may be experiencing more racial discrimination during the pandemic compared with other ethnic/racial groups in the United States. However, no research exists to analyze the rise of online discrimination of Asian Americans compared with online discrimination directed toward other racial groups. Therefore, we expect that, during the COVID-19 pandemic, individuals of Asian ethnicity would report experiencing more individual and vicarious online racial discrimination compared with individuals of non-Asian ethnicity (ie, White Americans, Black Americans) in the United States.

Prior to the COVID-19 pandemic, much research has examined the impact of racial discrimination on the mental health of ethnic minorities [38,54-64]. For example, Tynes et al [37] found that Black students and other adolescents of color were subjected to both vicarious and individual forms of online racial discrimination, which impacted students’ psychological functioning. In addition, both online vicarious and individual discrimination were significantly associated with worse psychological well-being among adults of racial/ethnic minorities (eg, Black Americans, Latinx Americans, Asian Americans) [34,35,39,40]. To our knowledge, existing research has only focused on the impacts of offline forms of racial discrimination in the context of COVID-19. Therefore, it remains unclear if online COVID-19 racial discrimination would also impact individuals’ mental health. Our study thus also examines how the online vicarious and individual racial discrimination individuals experienced during the COVID-19 pandemic might be associated with their anxiety, depression, and STS. Online racism has impacted the mental health outcomes of various racial/ethnic groups in the United States. However, as discussed earlier, this pandemic has been a particularly difficult time for Asian Americans as anti-Asian hate has been reported to be at an all-time high. Therefore, we also aim to assess whether individuals of Asian ethnicity would report higher negative mental health outcomes (anxiety, depression, and STS) compared with individuals of non-Asian ethnicity (eg, White Americans) in the United States.

### Mechanisms Underlying the Impact of Online Racial Discrimination on Mental Health

To understand the mechanisms underlying the impact of online racial discrimination on individuals’ mental health during the COVID-19 pandemic, we draw upon the Differential Susceptibility to Media Effects Model (DSMM) [65]. The DSMM is a contemporary communication-based model that describes the relationships between media use and various mental health outcomes. From the perspective of the DSMM, social media use can have an adverse effect on users’ cognition and emotional state and can produce negative physiological and behavioral outcomes [65]. Besides, individual differences such as race, gender, and ethnicity can moderate the strength of social media’s effects. There are 3 response states (cognitive, executive, and excitative states) that explain the connection between the use of online media and its outcomes [65]. For our study, we investigate racial/ethnic identification as a moderator and negative affect, an excitatory response state, as a mediator.

In this study, we define racial/ethnic identification as an amalgamation of racial and ethnic identities. Racial identification refers to the “multidimensional construct that includes the strength of one’s identification with one’s racial group, a sense of attachment to other group members, an evaluation of group membership” (eg, how much the individual likes or dislikes being White, for example) and “may include group-relevant attitudes and behaviors” [66]. Ethnic identification relates to a dynamic, social construct that is reflective of cultural practices as well as the acquisition and maintenance of cultural characteristics (eg, ethnic group behaviors, knowledge and awareness of cultural beliefs, and traditions of one’s ethnic group) [67-69]. Although these are distinct social constructs, they overlap in many ways, specifically regarding how racial/ethnic identification can help can help those who have experienced racialized discrimination better understand their experience as a communal rather than a personal one [70].

Findings from past research vary in terms of whether racial/ethnic identification should be considered as a buffer [71,72] or an exacerbator of the impact of racial discrimination on negative mental health outcomes [73-75]. For example, Tynes et al’s [76] study of Black adolescents found that ethnic identification moderated the negative impact of online racial discrimination on anxiety levels such that ethnic identification lessened the impact of discrimination on mental health. Although past research has shown that ethnic/racial identification may buffer against the negative impacts of racial discrimination [77], a recent meta-analysis revealed that the directionality of moderation varies for each ethnic/racial group [78]. Again, because past research has focused on offline forms of racial discrimination, no empirical research has examined whether the impact of online racial discrimination on individuals’ mental health outcomes varies as a result of an individual’s level of racial/ethnic identification. Therefore, we also examine the moderating role of racial-ethnic identification in the relationship between online racial discrimination and mental health during the COVID-19 pandemic.

Negative affect has been shown to be significantly associated with racial discrimination [79] and serves as a mediator between experiences of racial discrimination and health and psychological well-being [80,81]. For instance, Gibbons et al [81], as well as Gibbons and Stock [82], investigated the relationship between perceived racial discrimination and substance abuse, finding that negative affect mediated this relationship, such that increased perceived racial discrimination leads to greater experienced negative affect, which was positively associated with substance abuse [82,83]. Negative affect has also been shown to mediate the relationship between racial discrimination and other outcome variables such as delinquency [83] and reactive aggression for African American youth [84]. Recent research also found that social media usage concerning COVID-19 content and its effects on mental health was mediated by negative affect in a population of Chinese college students [85]. No study, however, has examined whether negative affect may mediate the impact of specific forms of online racial discrimination on psychological health. Therefore, our study also aims to assess whether negative affect can explain the effect of online racial discrimination on mental health outcomes.

## Methods

### Participants

The study was conducted through a university research participation portal as well as Amazon Mechanical Turk (MTurk) from March 3 to 15, 2021. Only individuals who were 18 years or older and were residing in the United States at the time of recruitment were qualified to participate in the study. Qualified participants recruited from the university research participation portal received course credit. Participants recruited from Amazon MTurk received a monetary compensation of US $0.50.

### Measures

#### Negative Affect

The 10‐item Negative Affect Scale of the Positive and Negative Affect Schedule was used to measure participants’ negative affect over the previous month [86]. Participants’ experience with negative affect was measured with a 5‐point Likert‐type scale (1=not really; 5=nearly every day). Participants were asked how frequently they experienced emotions/feelings such as “irritable,” “distressed,” and “afraid.” Total scores were averaged for analyses, with higher scores on the scale indicating greater negative affect (mean 2.26, SD 0.92; Cronbach α=.925).

#### Anxiety

An adapted version of the 7-item Generalized Anxiety Disorder Scale was used to measure participants’ anxiety during the previous month [87]. A 5-point Likert-type scale asked participants to respond on a scale from 1 (not at all) to 5 (nearly every day). Example items include “Not being able to stop or control worrying” and “Feeling afraid something awful might happen.” The 7 items were averaged to indicate the level of anxiety (mean 2.55, SD 1.10; Cronbach α=.932).

#### Depression

The 9-item Patient Health Questionnaire depression module [88] was used to measure participants’ depressive symptoms during the previous month. The scale consists of 9 items measured on a 5‐point Likert‐type graded response (1=not at all; 5=nearly every day). Example items include “Had little interest or pleasure in doing things” and “Had thoughts that you would be better off dead or thoughts of hurting yourself in some way.” The 9 items were averaged to indicate the level of depression (mean 2.37, SD 0.99; Cronbach α=.915).

#### Secondary Traumatic Stress

An adapted version of the 17-item Secondary Traumatic Stress Scale was used to measure participants’ STS during the previous month [89]. The scale assessed participants’ traumatic symptoms such as avoidance, intrusion, and arousal during the COVID-19 pandemic using 5‐point Likert‐type items (1=not at all; 5=nearly every day). Example items include “It seems as if I can relive the trauma(s) experienced through the pandemic” and “I wanted to avoid thinking about the pandemic.” All 17 items were averaged to indicate STS level (mean 2.30, SD 0.91; Cronbach α=.939).

#### Social Media Usage

Social media usage was measured with 6 items adapted from the social media scale developed by Yang et al [90]. The scale measures participants’ engagement in both active and passive use of social media on a scale of 1 (not at all) to 5 (regularly). Example questions include “How frequently have you paid attention to and read posts related to the COVID-19 pandemic on social media?” and “How frequently have you posted or reposted information and news related to the COVID-19 pandemic?” The 6 items were averaged to create an index of social media exposure (mean 2.33, SD 0.88; Cronbach α=.859).

#### Online Racial Discrimination

Participants’ experience of online racial discrimination during the pandemic was measured utilizing items adapted from the Online Victimization Scale [91]. A total of 5 items were adapted to measure online individual racial discrimination (eg, “I have been harassed or bothered online because of my race or ethnic group online”), and 5 items were adapted to measure online vicarious racial discrimination (eg, “People have said negative things [like rumors or name-calling] about my race or ethnic group online”). All items were measured with a 5-point Likert scale (1=never; 5=regularly) for each question. Responses to the 5 items regarding individual discrimination were averaged to create an index of individual online racial discrimination (mean 1.47, SD 0.95; Cronbach α=.962), and the overall level of vicarious racial discrimination online was calculated by averaging the 5 items for vicarious discrimination (mean 2.27, SD 1.22; Cronbach α=.941).

#### Racial/Ethnic Identification

Racial/ethnic identification was measured with an adapted version of the Asian American Identity Scale [92]. The Asian American Identity Scale was chosen due to its ability to address attachment and belonging to one’s racial/ethnic group, as well as pride in and awareness of prejudice against one’s racial/ethnic group. The original scale consists of 12 items, and items that referred specifically to Asian American identity or stereotypes were removed and the item “my racial/ethnic identity is important to me,” was added. Participants responded using a 7-point Likert-type scale (1=strongly disagree; 7=strongly agree). Example questions include “I feel a lot of pride in the achievements of my group,” “I try to carry out at least some of my group's customs and traditions (eg, relating to holidays, food, language),” and “It is important for me to learn about my group’s traditions, customs, and values.” Participants were asked to respond to the items based on their experiences over the last 6 months. Responses to the 7 individual items were averaged to indicate the level of racial identification (mean 4.93, SD 1.40; Cronbach α=.913).

#### Covariates

Several variables were also measured as covariates in this study. Specifically, mental health history was assessed by asking whether participants had a history of mental disorders prior to COVID-19. Participants responded either “Yes,” “No,” or “Prefer not to say.” Demographic characteristics including age, gender, level of education, and ethnicity were also assessed. Zhao and Zhou’s [85] 11-item COVID-19 stressor checklist tool was used to measure COVID-19–related stressors [85]. Participants were asked to respond “yes” or “no” to the following items: whether they experienced lockdown; known someone with confirmed or suspected (COVID-19) infection; experienced the death of a loved one(s) due to COVID-19; known an acquaintance dying from the COVID-19 infection; worked in a frontline job/as essential worker; interacted with infectious patients who had COVID-19; worked as an essential worker during this pandemic; volunteered in epidemic prevention and control; and lacked necessities such as food, face masks, disinfectants, and medical care. The average values and standard deviations of each of the variables measured are summarized in Multimedia Appendix 1.

### Ethics Approval

This study was approved by the institutional review board of the University of California, Davis (approval number: 1701406-1). All ethical procedures were maintained and followed, including the process of preserving web-based data privacy and security for data under the institutional review board protocol. This study abided by all applicable laws, regulations, and standard operations governing the protection of human subjects, student information, and protected health information. Participation in this study was completely voluntary. Written consent to collect data was obtained from all participants electronically and ensured that their identity would remain anonymous.

## Results

### Participants

A total of 1147 participants were included in the analysis after removing 35 incomplete responses, those who spent greater than 2 hours (n=11) or less than 5 minutes (n=297) on the survey, and those who incorrectly answered at least 1 of 4 attention checks (n=216). Of the total participants (n=1147), 676 (58.94%) were recruited through MTurk and 471 (41.06%) were undergraduates recruited from the researchers’ institution. Participants ranged in age from 18 to 80 years (mean 32, SD 14 years), and most were female (701/1147, 61.12%), with 423 (36.88%) identifying as male and 15 (1.31%) identifying as nonbinary. A total of 549 (47.86%) participants reported their race as White, 306 (26.68%) as Asian/Pacific Islander or mixed Asian, 133 (11.60%) as Latinx, 109 (9.50%) as Black, 11 (0.96%) as Indigenous, and 33 (2.88%) as mixed (not Asian). Asian American and Pacific Islander participants were combined for analysis because these groups have historically been studied in tandem [93] and have been shown to experience similar increases in instances of discrimination during the COVID-19 pandemic [51,53]. Additional demographic information is provided in Table 1.

**Table 1:** Participant demographics (N=1147).

Demographics						Values
Age (years), mean (SD)						32 (14.4)
**Gender, n (%)**						
	Male				423 (36.88)	
	Female				701 (61.12)	
	Nonbinary				15 (1.31)	
	Prefer not to say				4 (0.35)	
	Other				4 (0.35)	
**Race, n (%)**						
	Black				109 (9.50)	
	Latinx				133 (11.60)	
	Indigenous				11 (0.96)	
	AAPI^a^				306 (26.68)	
	White				549 (47.86)	
	Mixed				33 (2.88)	
**Asian ethnicity^b^, n (%)**						
	Chinese				117 (10.20)	
	Japanese				10 (0.87)	
	Korean				25 (2.18)	
	Filipino				27 (2.35)	
	Vietnamese				20 (1.74)	
	Indian				42 (3.66)	
	Pakistani				14 (1.22)	
	Bangladeshi				3 (0.26)	
	Sri Lankan				2 (0.17)	
	Hmong				3 (0.26)	
	Not shown				22 (1.92)	
	Mixed				15 (1.31)	
**Mechanical Turk sample (n=676)**						
	**Education, n (%)**					
		Less than high school		5 (0.44)		
		High school		48 (4.18)		
		Some college		91 (7.93)		
		Associates		74 (6.45)		
		Bachelors		304 (26.50)		
		Masters		137 (11.94)		
		Doctoral		12 (1.05)		
		Professional		5 (0.44)		
	**Employment, n (%)**					
		Self-employed		91 (7.93)		
		Full-time		396 (34.52)		
		Part-time		76 (6.63)		
		Out of work		32 (2.79)		
		Not able to work		14 (1.22)		
		Retired		36 (3.14)		
		Other		31 (2.70)		
	**Income (US $), n (%)**					
		<10,000		27 (2.35)		
		10,000-20,000		52 (4.53)		
		20,001-40,000		137 (11.94)		
		40,001-60,000		146 (12.73)		
		60,001-80,000		122 (10.64)		
		80,001-100,000		70 (6.10)		
		100,001-120,000		55 (4.80)		
		>120,000		67 (5.84)		
**College** **student** **sample (n=471)**						
	**College year, n (%)**					
			1	155 (13.51)		
			2	103 (8.98)		
			3	119 (10.37)		
			4	80 (6.97)		
			5	14 (1.22)		
	**Major, n (%)**					
			Social science	246 (21.45)		
			Humanities	21 (1.83)		
			Math/engineering	22 (1.92)		
			Biological science	125 (10.90)		
			Physical science	7 (0.61)		
			Undeclared	18 (1.57)		
			Professional school	1 (0.09)		

^a^AAPI: Asian American, Pacific Islander, and Mixed Race Asian identities (see Multimedia Appendix 2 for a more in-depth summary of the Asian ethnic subgroups).

^b^A total of 300 participants noted their Asian ethnicity.

### Predictors of Mental Health Outcomes

Multiple hierarchical regression models were run utilizing SPSS version 27 (IBM Corp) to assess social media usage and individual and vicarious discrimination as predictors of mental health outcomes. Step 1 included the covariates of mental health history, the experience of COVID-19 stressors, age, gender, race, education, and income. The independent variables of interest (COVID-19–related social media use, individual online discrimination, and vicarious online discrimination) were added in step 2. Full results from the hierarchical regressions are presented in Multimedia Appendices 3-5. The results showed that COVID-19–related social media use was positively associated with STS (β=.39, *P*<.001), depression (β=.36, *P*<.001), and anxiety (β=.30, *P*<.001). Individual online perceived discrimination was a significant positive predictor of STS (β=.52, *P*<.001), depression (β=.53, *P*<.001), and anxiety (β=.41, *P*<.001). Similarly, vicarious online perceived discrimination was positively associated with STS (β=.39, *P*<.001), depression (β=.39, *P*<.001), and anxiety (β=.33, *P*<.001).

### Racial/Ethnic Differences

To test whether Asian participants differed from other racial/ethnic groups in reports of individual and vicarious online perceived discrimination and negative mental health outcomes, a 1-way ANOVA and Bonferroni post hoc test were utilized. Black, Latinx, White, and AAPI groups were compared in the analysis. Because of the small sample size, Indigenous (n=11) and mixed (n=33) racial/ethnic groups were not included. There was a statistical difference between racial/ethnic groups for individual (*F*_3,1083_=31.23, *P*<.001; η^2^=0.080) and vicarious discrimination (*F*_3,1085_=46.40, *P*<.001; η^2^=0.114). Black participants (mean 2.21, SD 1.31) reported significantly higher individual discrimination than Asian (mean 1.34, SD 0.79; *P*<.001), Latinx (mean 1.13, SD 0.43; *P*<.001), and White (mean 1.47, SD 0.97; *P*<.001) participants. Additionally, Black participants (mean 2.98, SD 1.18; *P*<.001) reported significantly higher vicarious discrimination than Latinx (mean 2.24, SD 0.97; *P*<.001) and White (mean 1.90, SD 1.14; *P*<.001) participants. Asian participants reported significantly higher vicarious discrimination (mean 2.69, SD 1.23) than Latinx (mean 2.23, SD 0.97; *P*<.001) and White participants (mean 1.90, SD 1.14; *P*<.001). Therefore, partial support for Asian Americans reporting higher discrimination was found.

There was a statistical difference between racial/ethnic groups for depression (*F*_3,1068_=6.73, *P*<.001; η^2^=0.019) and anxiety (*F*_3,1076_=3.40, *P*=.017; η^2^=.009) but not for STS. Post hoc analyses showed that AAPI participants did not differ significantly in STS from White (*P*>.99), Black (*P*>.99), or Latinx (*P*=.97) participants. AAPI participants also did not differ significantly in depression from White (*P*=.05), Black (*P*>.99), or Latinx (*P*=.23) participants or differ significantly in anxiety from White (*P*=.42), Black (*P*>.99), or Latinx (*P*=.80) participants.

Considering that Chinese participants made up the largest ethnic subgroup in the sample of Asian participants, an unpaired independent sample *t* test (2-tailed) was used to determine whether Chinese participants experienced different levels of discrimination and mental health outcomes compared with other Asian ethnicities. Chinese participants experienced lower levels of STS (n=114; mean 1.99, SD 0.75) compared with other Asian participants (n=185; mean 2.48, SD 0.91; t_274.78_=–5.016, *P*<.001). Chinese participants also experienced lower levels of depression (n=114; mean 2.18, SD 0.87) compared with other Asian participants (n=187; mean 2.61, SD 0.99; t_262.63_=–3.991, *P*<.001) and anxiety (n=115; mean 2.30, SD 0.98) compared with other Asian participants (n=188; mean 2.61, SD 0.99; t_271.828_=–3.978, *P*<.001). Lastly, other Asian participants (n=187; mean 2.84, SD 1.24) experienced more vicarious discrimination compared with Chinese participants (n=117; mean 2.46, SD 1.19; t_253.02_=–2.692, *P*=.008).

### Racial/Ethnic Identification

To address the moderating role of racial/ethnic identification in the relationship between individual and vicarious online racial discrimination and the 3 mental health outcomes, the interaction between individual and vicarious online racial discrimination and racial/ethnical identification was tested utilizing model 1 of the PROCESS macro for SPSS by Hayes [94]. Racial/ethnic identification was a significant moderator between both individual (β=.07, *P*=.01) and vicarious (β=.06, *P*=.002) online racial discrimination and STS such that the association between discrimination and STS was stronger as racial identity increased. This was not the case for depression or anxiety for either type of discrimination.

### Negative Affect

Lastly, the role of negative affect as a mediator in the relationship between individual and vicarious discrimination and the 3 mental health outcomes was assessed using model 4 of the PROCESS macro. Negative affect mediated the effects of individual discrimination on STS (indirect effect [IE] 0.3375, 95% CI 0.27-0.42) and depression (IE 0.3332, 95% CI 0.26-0.41), and fully mediated the effects of individual discrimination on anxiety (IE 0.3974, 95% CI 0.31-0.49). Negative affect also mediated the effects of vicarious discrimination on STS (IE 0.2379, 95% CI 0.18-0.31) and depression (IE 0.2369, 95% CI 0.18-0.30) and fully mediated the effects of individual discrimination on anxiety (IE 0.2711, 95% CI 0.21-0.35).

## Discussion

### Principal Findings

The main goal of this study was to examine the impact of social media use and racial discrimination on individuals’ mental health (ie, symptoms of depression, anxiety, and STS) among different ethnic and racial groups in the United States during the COVID-19 pandemic. In addition, we investigated the moderating effects of racial/ethnic identification and the mediating role of negative affect in those relationships.

The findings of our study support several general conclusions. First, as expected, we found that increased use of social media during the public health crisis of the COVID-19 pandemic had a negative impact on users’ mental health. This finding resonates with those from similar studies that have examined social media usage and its impact on people’s mental health during the pandemic in other countries such as China [9,24,95], Bolivia [96], and Bangladesh [97].

This finding holds important pragmatic implications. Although media can provide social connectivity and public health updates, it can also contribute to the negative mental well-being of users. Social media has fueled the rapid spread of misinformation (eg, false news and racial discrimination) during this pandemic [98-101], which may create a sense of panic and confusion, leading to problematic social media usage (PSMU)—a form of addiction to social media that is associated with anxiety, depressive symptoms, and low self-esteem [102]. There is evidence that PSMU has increased during the pandemic, that is, when social isolation became the new norm [103-106]. Although this study did not directly examine PSMU, it is likely that the observed negative association between social media use specifically related to COVID-19 content and mental health outcomes can be partially attributed to PSMU. Future research should explore the relationship between PSMU and COVID-19–related social media use and their impact on mental health.

The connection between social media usage related to the COVID-19 pandemic and negative mental health outcomes shows the importance not only of further research into the topic, but also the need for actions such as policy change and educational campaigns [9,107]. In particular, concrete recommendations may be introduced and encouraged to combat misinformation and PSMU. For instance, social media platforms can reinforce more website policing to delineate false online information and regulate the content. With negative information being regulated, this may mitigate the negative media effects on users. Users may also seek fact-checking labels on posts as well as other sources of news to verify online statements. Additionally, national policy may be implemented to further limit and moderate social media content that includes hateful speech and discriminatory online behaviors. In addition, social media users may want to reduce their screen time and actively become mindful consumers of media. Online users can use various methods such as apps to track their screen time or disabling notifications to limit usage. Although placing the onus on individual social media users is not the only approach to reducing the negative impacts of social media use, recent studies have demonstrated that behavioral, self-imposed regulations may be powerful methods to regulate usage and promote healthy online habits. Additionally, future long-term strategies have included the promotion of digital health guidelines that are taught in public sectors and enforced by evidence-based policy and legislation [101].

Consistent with our predictions, individual and vicarious forms of online discrimination experienced during the COVID-19 pandemic were found to be positively associated with STS, depression, and anxiety. Our data also revealed several interesting findings regarding different ethnic groups’ experiences during the pandemic. On the one hand, Asian participants in our study reported significantly higher vicarious discrimination than both Latinx and White participants. This finding corroborates recent research concerning discrimination experienced by Asian Americans [53,73,90]. In relation to our large Chinese subpopulation, we also found that Chinese participants experienced lower levels of each of the mental health outcomes and lower levels of vicarious discrimination compared with other Asian participants, which reflects the rise in online hate toward the group of Asian Americans as a whole [53]. On the other hand, Asian American participants in our study also did not perceive significantly more individual online discrimination compared with the other racial/ethnic groups. Although this study did not compare sources of discrimination, previous research suggests that this finding may be related to the fact that multiple racial/ethnic minority communities have experienced distinct forms of discrimination during this pandemic in the spheres of health care, accessibility to resources, and vaccines, and more [108-111]. Research on Black Americans, for example, points to structural health discrimination and bioethical concerns causing the “epidemic” of premature black death [112-114]. Additionally, there is evidence showing that Indigenous and Latinx groups had the highest hospitalization and mortality rates in the United States during the pandemic [115-119].

Contrary to our expectations, in comparison to other large racial groups in our study, Black Americans reported experiencing more online discrimination compared with Asian Americans. In particular, we found that Black Americans reported the highest levels of individual as well as vicarious discrimination among all the racial/ethnic groups examined in this study, corresponding to the vast prevalence of online hatred directed toward Black Americans throughout US history [60,120-122]. These findings may be related to the collective hardships experienced by Black Americans, who continued to experience heightened forms of hate and systemic oppression made salient during the COVID-19 pandemic through media coverage and social movements such as Black Lives Matter [123-125].

Black Americans also reported the worst mental health outcomes. As a result, this study emphasizes the importance of recognizing the mental health struggles of this community, especially due to the disproportionate number of deaths in the Black community from COVID-19 health disparities [126]. Additionally, racism and bias do impact medical care, as research shows that Black women are much more likely to not receive appropriate diagnoses for depression, and are more likely to be seen as having psychosis than depression [127].

The findings from our study also revealed that racial/ethnic identification moderates both individual and vicarious discrimination for STS. In other words, it can be understood that the STS levels of individuals who identify more strongly with their racial/ethnic community are more likely to be impacted by their experience with individual and vicarious discrimination. If one closely aligns themself and takes pride in their racial/ethnic community, attacks on that identity or on others who share that identity would be more personally devastating to them than to someone who is not as aligned with their racial/ethnic community. Considering that the extent to which one identifies with their racial/ethnic identity can be a powerful indicator of how adversely racial discrimination can impact their mental health outcomes, certain individuals may benefit more from reducing their exposure to online content or taking steps toward limiting their mental health effects of social media use by seeking mental health care. Racial/ethnic identification was not a moderator for the other 2 mental health outcomes. Taken as a whole, our findings regarding online racial discrimination suggest the necessity for some policy and educational interventions. For example, if policy-level regulations, such as content blocking, were implemented on a national level, there is a possibility that racial hate online could significantly decrease. Moreover, culturally sensitive educational plans for children and youth can be made to promote cross-cultural understanding and social awareness of social issues online. These potential educational opportunities that specifically discuss online racial discrimination and hate could be useful for reducing the prevalence of hateful speech on social media as well as providing social media users with ways to reduce the negative mental health impacts of online racial discrimination.

### Comparison With Prior Work

Our study connects with prior research in several ways. First, regarding the impact of social media use and discrimination on mental health, our findings are consistent with past research that shows that increases in media use are common during experiences of collective trauma [128,129]. Importantly, it echoes past findings regarding the impact of media usage related to trauma and disaster content on psychological well-being [95,130-136]. Additionally, our finding of increased racial discrimination being correlated with worse negative mental health outcomes is also in line with previous work [31,54-63]. Although our study did not distinguish between online racism and online discrimination, it aligns with recent studies, demonstrating that online discrimination, including online racism, during the pandemic could have a substantial negative impact on individuals’ mental health [34,35,38,39]. Second, we were intrigued to find that White Americans in our study reported experiencing more individual discrimination than Asian Americans during the COVID-19 pandemic. Previous research has shown that White Americans perceive different types of discrimination compared to those experienced by people of color and that White Americans report experiencing discrimination as a racial group [137-139]. This anti-White bias is empirically shown to be linked to worse mental wellness for White participants [137]. National surveys have indicated that White individuals have reported discrimination in several public sectors [138,139]. For instance, a national poll conducted by Harvard found that 19% of White Americans in its sample said that they have been personally discriminated against because they are White when applying for jobs, and 28% of White individuals who believe in the existence of anti-White discrimination reported that they have personally experienced hearing insensitive comments related to their race [139].

Finally, our findings suggest that negative affect could aid in explaining the relationship between online racial discrimination and mental health outcomes. In the past, online and offline experiences with racial discrimination have been shown to trigger certain negative emotional or physical responses (eg, stress), which in turn may lead to worse mental well-being. However, this was not explicitly examined as negative affect [36,140,141]. Our findings suggest that negative affect may explain how racial discrimination is linked to worsening behavioral [85] and psychological well-being [82,83] in offline and online contexts. This finding is also consistent with the pathway proposed by the DSMM, that is, negative emotions or affective states may explain the relationship between online racial discrimination on social media and negative mental health outcomes.

### Limitations and Recommendations for Future Research

A primary limitation of our study was the method used for data collection, specifically the use of college students as well as participants recruited from Amazon MTurk. Although the college student sample primarily identified as Asian or mixed Asian (232/471, 49.3%), other racial/ethnic groups, such as Black individuals (109/1147, 9.50%), were underrepresented in our sample; thus, experiences of discrimination online may have been underreported. Additionally, some studies indicate that MTurk samples may not be as representative as national probability samples [99,142,143]. Considering that we were unable to conduct a pan-ethnic survey and that the majority of the participants in our survey were White, it is reasonable to assume that if we opted for a different platform for data collection, our results may have been different and may have better represented the experience of all Americans [83]. Because of our limited sample size, we could not conduct a national survey. Our cross-sectional study was not able to analyze and compare all experiences of individuals from all race/ethnic groups in the US population.

Another limitation of our study was the time frame for which we kept the survey open. At the time of data collection, the United States was approaching an end to strict COVID-19 guidelines, and data collection was completed in 2 weeks in consideration of the rapidly evolving nature of the pandemic. However, the time frame during which we collected our data was not during the peak of the pandemic, nor the peak of racialized discrimination against Asian Americans. As such, we believe that the true salience of discrimination against AAPI was not fully captured by this survey, as many hate crimes were reported taking place in 2020 compared with the time of data collection [53].

Because of the limited size and representativeness of our sample, we were unable to run pan-ethnic comparisons of various Asian ethnicities (eg, comparing Japanese Americans with Indian Americans). Future research should conduct an examination of pan-ethnic comparisons of various Asian ethnicities, which will provide more insights into the experiences of Asian American communities. As previously mentioned, due to our data collection methods and inability to keep our survey available for longer periods of data collection, we could not examine changes in perceived online discrimination or the longer effects of exposure to online discrimination. Therefore, it remains important for future research to capture how changes in the prevalence of online discrimination in the wake of global events impact racial/ethnic minority groups.

### Conclusions

Our study demonstrated a high prevalence of mental health concerns associated with social media usage related to COVID-19, as well as a link between perceived online discrimination and poor mental health during the pandemic. Our results concerning online social media may reflect the offline experiences and struggles of several racial/ethnic communities. Our study used a self-report survey data collection method to examine Asian Americans and various racial/ethnic groups living in the United States as they experienced perceived discrimination during the pandemic. This research and additional studies should broaden the scientific community’s and the public’s understanding of different ethnic groups’ social media use and its impact on their mental health, especially during a period of crisis.

## Appendix

Multimedia Appendix 1 Summary of independent and dependent variables.

Multimedia Appendix 2 Summary of Asian ethnicities.

Multimedia Appendix 3 Ordinal logistic regression results for secondary traumatic stress.

Multimedia Appendix 4 Ordinal logistic regression results for depression.

Multimedia Appendix 5 Ordinal logistic regression results for anxiety.

## Abbreviations

AAPI: Asian, Pacific Islander, and Mixed Asian
DSMM: Differential Susceptibility to Media Effects Model
IE: indirect effect
MTurk: Mechanical Turk
PSMU: problematic social media usage
STS: secondary traumatic stress
