# Supplementary material for: The Impacts of Social Media Use and Online Racial Discrimination on Asian American Mental Health: Cross-sectional Survey in the United States During COVID-19
Source: JMIR Form Res. 2022 Sep 19;6(9):e38589. doi: 10.2196/38589 (PMC9488547; doi:10.2196/38589)
Supplement: Multimedia Appendix 1 [file formative_v6i9e38589_app1.docx]

Table S1. Summary of Independent and Dependent Variables

|  | *n* | *M* | *SD* |
| --- | --- | --- | --- |
| **COVID-19 Stressors** | 1130 | .37 | .21 |
| AAPI^s^ | 303 | .32 | .19 |
| White | 543 | .37 | .22 |
| Black | 103 | .41 | .24 |
| Latinx | 132 | .42 | .17 |
| **Social Media Use** | 1133 | 2.33 | .88 |
| AAPI | 303 | 2.32 | .82 |
| White | 542 | 2.23 | .89 |
| Black | 107 | 2.76 | .93 |
| Latinx | 133 | 2.37 | .89 |
| **Individual Discrimination** | 1136 | 1.47 | .95 |
| AAPI | 302 | 1.34 | .79 |
| White | 546 | 1.47 | .97 |
| Black | 106 | 2.21 | 1.31 |
| Latinx | 133 | 1.13 | .43 |
| **Vicarious Discrimination** | 1138 | 2.27 | 1.22 |
| AAPI | 304 | 2.69 | 1.23 |
| White | 545 | 1.90 | 1.14 |
| Black | 109 | 2.98 | 1.18 |
| Latinx | 131 | 2.24 | .97 |
| **Negative Affect** | 1124 | 2.26 | .92 |
| AAPI | 299 | 2.34 | .91 |
| White | 542 | 2.19 | .93 |
| Black | 106 | 2.24 | 1.07 |
| Latinx | 129 | 2.40 | .81 |
| **Racial/Ethnic Identification** | 1134 | 4.93 | 1.40 |
| AAPI | 301 | 5.55 | 1.00 |
| White | 545 | 4.22 | 1.37 |
| Black | 106 | 5.69 | 1.10 |
| Latinx | 133 | 5.82 | 1.07 |
| **STS** | 1110 | 2.30 | .91 |
| AAPI | 300 | 2.30 | .88 |
| White | 530 | 2.25 | .92 |
| Black | 105 | 2.37 | 1.04 |
| Latinx | 127 | 2.43 | .82 |
| **Depression** | 1120 | 2.37 | .99 |
| AAPI | 301 | 2.45 | .97 |
| White | 539 | 2.26 | .99 |
| Black | 104 | 2.30 | 1.09 |
| Latinx | 128 | 2.66 | .92 |
| **Anxiety** | 1130 | 2.55 | 1.10 |
| AAPI | 303 | 2.60 | 1.12 |
| White | 540 | 2.46 | 1.08 |
| Black | 109 | 2.48 | 1.20 |
| Latinx | 128 | 2.78 | 1.05 |
|  | Yes | No |  |
| **Mental Health History^b^** | 288 | 815 |  |
| AAPI | 51 | 242 |  |
| White | 177 | 354 |  |
| Black | 20 | 85 |  |
| Latinx | 27 | 100 |  |

^a^ AAPI = Asian, Pacific Islander and Mixed Asian identities

^b^ Participants were asked if they had been diagnosed with a mental illness prior to the COVID-19
