# Supplementary material for: The Impacts of Social Media Use and Online Racial Discrimination on Asian American Mental Health: Cross-sectional Survey in the United States During COVID-19
Source: JMIR Form Res. 2022 Sep 19;6(9):e38589. doi: 10.2196/38589 (PMC9488547; doi:10.2196/38589)
Supplement: Multimedia Appendix 2 [file formative_v6i9e38589_app2.docx]

Table S2. Summary of Asian Ethnicities

|  | *n* | *% of Asian Sample (n=306)* | *% of Total Sample (n=1147)* |
| --- | --- | --- | --- |
| Chinese | 117 | 38.2 | 10.2 |
| Japanese | 10 | 3.3 | .9 |
| Korean | 25 | 8.2 | 2.2 |
| Filipino | 27 | 8.8 | 2.4 |
| Vietnamese | 20 | 6.5 | 1.7 |
| Indian | 42 | 13.7 | 3.7 |
| Pakistani | 14 | 4.6 | 1.2 |
| Bangladeshi | 3 | 1 | .3 |
| Sri Lankan | 2 | .7 | .2 |
| Hmong | 3 | 1 | .3 |
| Mixed | 15 | 4.9 | 1.3 |
| Not Shown | 22 | 7.2 | 1.9 |
| Did Not Report Ethnicity | 6 | 2 | .5 |
