# Supplementary material for: The Impacts of Social Media Use and Online Racial Discrimination on Asian American Mental Health: Cross-sectional Survey in the United States During COVID-19
Source: JMIR Form Res. 2022 Sep 19;6(9):e38589. doi: 10.2196/38589 (PMC9488547; doi:10.2196/38589)
Supplement: Multimedia Appendix 3 [file formative_v6i9e38589_app3.docx]

Table S3. Ordinal Logistic Regression Results for Secondary Traumatic Stress

|  |  |  |  | |  | |  | H1. Social Media Use | | |
| --- | --- | --- | --- | --- | --- | --- | --- | --- | --- | --- |
|  |  | Step 1 | | | | |  | Step 2 | | |
| Predictors | | *B* | | *SE* | | β |  | *B* | *SE* | β |
| MH History | | .58 | | .07 | | .27^***^ |  | .50 | .07 | .23^***^ |
| COVID Stressors | | 1.96 | | .14 | | .46^***^ |  | 1.29 | .15 | .30^***^ |
| Age | | -.01 | | .002 | | -.1^**^ |  | -.01 | .002 | -.08^*^ |
| **Gender** | |  | |  | |  |  |  |  |  |
|  | Male^a^ | .02 | | .07 | | .01 |  | .01 | .06 | .01 |
|  | Other | -.23 | | .31 | | -.02 |  | -.27 | .28 | -.03 |
| **Race/Ethnicity** | |  | |  | |  |  |  |  |  |
|  | Black^b^ | .09 | | .10 | | .03 |  | -.11 | .09 | -.04 |
|  | Latinx | -.11 | | .18 | | -.02 |  | -.04 | .16 | -.007 |
|  | AAPI^c^ | .10 | | .11 | | .03 |  | .12 | .10 | .04 |
|  | Other | .05 | | .17 | | .01 |  | .05 | .16 | .01 |
| Education | | .06 | | .03 | | .08^*^ |  | .02 | .03 | .02 |
| Income | | -.03 | | .02 | | -.05 |  | -.02 | .02 | -.04 |
| Social Media Use | |  | |  | |  |  | .41 | .04 | .39^***^ |
| Individual Discrimination | |  | |  | |  |  |  |  |  |
| Vicarious Discrimination | |  | |  | |  |  |  |  |  |
| *R*^2^ | |  | | .380 | |  |  |  | .483 |  |

|  |  | H2. Individual Discrimination | | |  | H3. Vicarious Discrimination | | |
| --- | --- | --- | --- | --- | --- | --- | --- | --- |
|  |  | Step 2 | | |  | Step 2 | | |
| Predictors | | *B* | *SE* | β |  | *B* | *SE* | β |
| MH History | | .44 | .06 | .21^***^ |  | .49 | .07 | .23^***^ |
| COVID Stressors | | .70 | .16 | .16^***^ |  | 1.29 | .15 | .30^***^ |
| Age | | -.01 | .002 | -.08^**^ |  | -.01 | .002 | -.06^*^ |
| **Gender** | |  |  |  |  |  |  |  |
|  | Male^a^ | -.14 | .06 | -.07^*^ |  | -.07 | .06 | -.03 |
|  | Other | -.17 | .27 | -.02 |  | -.25 | .28 | -.03 |
| **Race/Ethnicity** | |  |  |  |  |  |  |  |
|  | Black^b^ | -.19 | .09 | -.07^*^ |  | -.17 | .09 | -.06 |
|  | Latinx | .02 | .16 | .003 |  | -.09 | .16 | -.02 |
|  | AAPI^c^ | .07 | .10 | .02 |  | -.06 | .10 | -.02 |
|  | Other | .004 | .15 | .001 |  | .01 | .16 | .001 |
| Education | | .003 | .02 | .004 |  | .03 | .03 | .04 |
| Income | | -.03 | .02 | -.05 |  | -.02 | .02 | -.04 |
| Social Media Use | |  |  |  |  |  |  |  |
| Individual Discrimination | | .47 | .04 | .52^***^ |  |  |  |  |
| Vicarious Discrimination | |  |  |  |  | .32 | .03 | .39^***^ |
| R^2^ | |  | .517 |  |  |  | .482 |  |

* *p* < .05, ** *p* < .01, *** p < .001

^a^ Reference group = Female

^b^ Reference group = White

^c^AAPI = Asian American, Pacific Islander, and Mixed Race Asian identities
