# Supplementary material for: The Impacts of Social Media Use and Online Racial Discrimination on Asian American Mental Health: Cross-sectional Survey in the United States During COVID-19
Source: JMIR Form Res. 2022 Sep 19;6(9):e38589. doi: 10.2196/38589 (PMC9488547; doi:10.2196/38589)
Supplement: Multimedia Appendix 4 [file formative_v6i9e38589_app4.docx]

Table S4. Ordinal Logistic Regression Results for Depression

|  |  |  |  | |  | |  | H1. Social Media Use | | |
| --- | --- | --- | --- | --- | --- | --- | --- | --- | --- | --- |
|  |  | Step 1 | | | | |  | Step 2 | | |
| Predictors | | *B* | | *SE* | | β |  | *B* | *SE* | β |
| MH History | | .70 | | .08 | | .30^***^ |  | .61 | .07 | .27^***^ |
| COVID Stressors | | 1.83 | | .15 | | .41^***^ |  | 1.15 | .16 | .26^***^ |
| Age | | -.01 | | .003 | | -.17^***^ |  | -.01 | .002 | -.15^***^ |
| **Gender** | |  | |  | |  |  |  |  |  |
|  | Male^a^ | .01 | | .07 | | .004 |  | .01 | .06 | .004 |
|  | Other | -.05 | | .32 | | -.01 |  | -.09 | .30 | -.01 |
| **Race/Ethnicity** | |  | |  | |  |  |  |  |  |
|  | Black^b^ | .00 | | .10 | | .00 |  | -.21 | .10 | -.07^*^ |
|  | Latinx | -.08 | | .19 | | -.01 |  | -.03 | .18 | -.01 |
|  | AAPI^c^ | .10 | | .11 | | .03 |  | .12 | .11 | .03 |
|  | Other | -.13 | | .18 | | -.02 |  | -.14 | .17 | -.02 |
| Education | | .02 | | .03 | | .02 |  | -.02 | .03 | -.03 |
| Income | | -.04 | | .02 | | -.06 |  | -.03 | .02 | -.05 |
| Social Media Use | |  | |  | |  |  | .41 | .04 | .36^***^ |
| Individual Discrimination | |  | |  | |  |  |  |  |  |
| Vicarious Discrimination | |  | |  | |  |  |  |  |  |
| *R*^2^ | |  | | .382 | |  |  |  | .467 |  |

|  |  | H2. Individual Discrimination | | |  | H3. Vicarious Discrimination | | |
| --- | --- | --- | --- | --- | --- | --- | --- | --- |
|  |  | Step 2 | | |  | Step 2 | | |
| Predictors | | *B* | *SE* | β |  | *B* | *SE* | β |
| MH History | | .55 | .07 | .24^***^ |  | .60 | .07 | .26^***^ |
| COVID Stressors | | .47 | .17 | .10^**^ |  | 1.09 | .16 | .24^***^ |
| Age | | -.01 | .002 | -.16^***^ |  | -.01 | .002 | -.14^***^ |
| **Gender** | |  |  |  |  |  |  |  |
|  | Male^a^ | -.14 | .06 | -.06^*^ |  | -.08 | .06 | -.04 |
|  | Other | .01 | .28 | .001 |  | -.07 | .30 | -.01 |
| **Race/Ethnicity** | |  |  |  |  |  |  |  |
|  | Black^b^ | -.28 | .09 | -.09^**^ |  | -.28 | .10 | -.09^**^ |
|  | Latinx | .04 | .17 | .007 |  | -.10 | .18 | -.02 |
|  | AAPI^c^ | .07 | .10 | .02 |  | -.07 | .11 | -.02 |
|  | Other | -.17 | .16 | -.03 |  | -.19 | .17 | -.03 |
| Education | | -.04 | .03 | -.05 |  | -.01 | .03 | -.01 |
| Income | | -.04 | .02 | -.06^*^ |  | -.03 | .02 | -.05 |
| Social Media Use | |  |  |  |  |  |  |  |
| Individual Discrimination | | .50 | .04 | .53^***^ |  |  |  |  |
| Vicarious Discrimination | |  |  |  |  | .35 | .03 | .39^***^ |
| R^2^ | |  | .522 |  |  |  | .488 |  |

* *p* < .05, ** *p* < .01, *** p < .001

^a^ Reference group = Female

^b^ Reference group = White

^c^AAPI = Asian American, Pacific Islander, and Mixed Race Asian identities
