# Supplementary material for: The Impacts of Social Media Use and Online Racial Discrimination on Asian American Mental Health: Cross-sectional Survey in the United States During COVID-19
Source: JMIR Form Res. 2022 Sep 19;6(9):e38589. doi: 10.2196/38589 (PMC9488547; doi:10.2196/38589)
Supplement: Multimedia Appendix 5 [file formative_v6i9e38589_app5.docx]

Table S5. Ordinal Logistic Regression Results for Anxiety

|  |  |  |  | |  | |  | H1. Social Media Use | | |
| --- | --- | --- | --- | --- | --- | --- | --- | --- | --- | --- |
|  |  | Step 1 | | | | |  | Step 2 | | |
| Predictors | | *B* | | *SE* | | β |  | *B* | *SE* | β |
| MH History | | .84 | | .08 | | .34^***^ |  | .76 | .08 | .30^***^ |
| COVID Stressors | | 1.73 | | .17 | | .35^***^ |  | 1.11 | .18 | .23^***^ |
| Age | | -.01 | | .003 | | -.17^***^ |  | -.01 | .003 | -.16^***^ |
| **Gender** | |  | |  | |  |  |  |  |  |
|  | Male^a^ | -.11 | | .08 | | -.05 |  | -.12 | .07 | -.05 |
|  | Other | -.14 | | .36 | | -.01 |  | -.16 | .34 | -.02 |
| **Race/Ethnicity** | |  | |  | |  |  |  |  |  |
|  | Black^b^ | -.04 | | .11 | | -.01 |  | -.23 | .11 | -.07^*^ |
|  | Latinx | -.32 | | .21 | | -.05 |  | -.25 | .20 | -.04 |
|  | AAPI^c^ | -.05 | | .13 | | -.01 |  | -.03 | .12 | -.01 |
|  | Other | .19 | | .19 | | .03 |  | .11 | .19 | .02 |
| Education | | -.01 | | .03 | | -.01 |  | -.05 | .03 | -.05 |
| Income | | -.04 | | .02 | | -.07^*^ |  | -.03 | .02 | -.05 |
| Social Media Use | |  | |  | |  |  | .37 | .05 | .30^***^ |
| Individual Discrimination | |  | |  | |  |  |  |  |  |
| Vicarious Discrimination | |  | |  | |  |  |  |  |  |
| *R*^2^ | |  | | .356 | |  |  |  | .411 |  |

|  |  | H2. Individual Discrimination | | |  | H3. Vicarious Discrimination | | |
| --- | --- | --- | --- | --- | --- | --- | --- | --- |
|  |  | Step 2 | | |  | Step 2 | | |
| Predictors | | *B* | *SE* | β |  | *B* | *SE* | β |
| MH History | | .70 | .08 | .28^***^ |  | .74 | .08 | .29^***^ |
| COVID Stressors | | .61 | .19 | .12^**^ |  | 1.10 | .18 | .22^***^ |
| Age | | -.01 | .003 | -.16^***^ |  | -.01 | .003 | -.14^***^ |
| **Gender** | |  |  |  |  |  |  |  |
|  | Male^a^ | -.24 | .07 | -.10^**^ |  | -.19 | .07 | -.08^*^ |
|  | Other | -.08 | .34 | -.01 |  | -.14 | .34 | -.01 |
| **Race/Ethnicity** | |  |  |  |  |  |  |  |
|  | Black^b^ | -.28 | .11 | -.08^*^ |  | -.30 | .11 | -.09^**^ |
|  | Latinx | -.18 | .20 | -.03 |  | -.32 | .20 | -.05 |
|  | AAPI^c^ | -.08 | .12 | -.02 |  | -.22 | .12 | -.06 |
|  | Other | .15 | .18 | .03 |  | .15 | .19 | .02 |
| Education | | -.06 | .03 | -.07^*^ |  | -.03 | .03 | -.03 |
| Income | | -.04 | .02 | -.07^*^ |  | -.04 | .02 | -.06 |
| Social Media Use | |  |  |  |  |  |  |  |
| Individual Discrimination | | .42 | .04 | .41^***^ |  |  |  |  |
| Vicarious Discrimination | |  |  |  |  | .31 | .04 | .33^***^ |
| R^2^ | |  | .437 |  |  |  | .431 |  |

* *p* < .05, ** *p* < .01, *** p < .001

^a^ Reference group = Female

^b^ Reference group = White

^c^AAPI = Asian American, Pacific Islander, and Mixed Race Asian identities
